# Association of single nucleotide polymorphisms with insulin secretion, insulin sensitivity, and diabetes in women with a history of gestational diabetes mellitus

**DOI:** 10.1186/s12920-021-01123-6

**Published:** 2021-11-20

**Authors:** Rashmi B. Prasad, Karl Kristensen, Anastasia Katsarou, Nael Shaat

**Affiliations:** 1 Genomics, Diabetes and Endocrinology, Department of Clinical Sciences, Lund University, Malmö, Sweden; 2 Department of Obstetrics and Gynaecology, Skåne University Hospital, Malmö, Sweden; 3 Department of Endocrinology, Skåne University Hospital, 205 02 Malmö, Sweden

**Keywords:** Prediction of diabetes, Gestational diabetes, Genetics of type 2 diabetes, Insulin sensitivity

## Abstract

**Background:**

This study investigated whether single nucleotide polymorphisms (SNPs) reported by previous genome-wide association studies (GWAS) to be associated with impaired insulin secretion, insulin resistance, and/or type 2 diabetes are associated with disposition index, the homeostasis model assessment of insulin resistance (HOMA-IR), and/or development of diabetes following a pregnancy complicated by gestational diabetes mellitus (GDM).

**Methods:**

Seventy-two SNPs were genotyped in 374 women with previous GDM from Southern Sweden. An oral glucose tolerance test was performed 1–2 years postpartum, although data on the diagnosis of diabetes were accessible up to 5 years postpartum. HOMA-IR and disposition index were used to measure insulin resistance and secretion, respectively.

**Results:**

The risk A-allele in the rs11708067 polymorphism of the adenylate cyclase 5 gene (*ADCY5*) was associated with decreased disposition index (beta = − 0.90, SE 0.38, *p* = 0.019). This polymorphism was an expression quantitative trait loci (eQTL) in islets for both *ADCY5* and its antisense transcript. The risk C-allele in the rs2943641 polymorphism, near the insulin receptor substrate 1 gene (*IRS1*), showed a trend towards association with increased HOMA-IR (beta = 0.36, SE 0.18, *p* = 0.050), and the T-allele of the rs4607103 polymorphism, near the ADAM metallopeptidase with thrombospondin type 1 motif 9 gene (*ADAMTS9*), was associated with postpartum diabetes (OR = 2.12, SE 0.22, *p* = 0.00055). The genetic risk score (GRS) of the top four SNPs tested for association with the disposition index using equal weights was associated with the disposition index (beta = − 0.31, SE = 0.29, *p* = 0.00096). In addition, the GRS of the four SNPs studied for association with HOMA-IR using equal weights showed an association with HOMA-IR (beta = 1.13, SE = 0.48, *p* = 9.72874e−11). All analyses were adjusted for age, body mass index, and ethnicity.

**Conclusions:**

This study demonstrated the genetic susceptibility of women with a history of GDM to impaired insulin secretion and sensitivity and, ultimately, to diabetes development.

## Background

During pregnancy, insulin sensitivity progressively decreases, while associated insulin response increases by late gestation [1]. Gestational diabetes mellitus (GDM) develops when beta-cells cannot compensate for increased insulin resistance, despite the physiological changes related to glucose homeostasis during gestation [1]. Women with a history of GDM have a higher risk of developing type 2 diabetes [2] and metabolic syndrome [3] than those who were normoglycaemic during pregnancy. Genetic risk factors related to progression to type 2 diabetes in women with a history of GDM have been previously studied by many research groups, including ours. Two polymorphisms (*TCF7L2* rs7903146 and *FTO* rs8050136) and a weighted risk score of type 2 diabetes risk alleles predict diabetes following GDM [4]. Other research groups have shown an association of the genetic risk score (GRS) with eventual progression to diabetes after GDM [5]. However, GRS could not predict the progression to diabetes in women with a history of GDM from the Diabetes Prevention Program (DPP) [6].

Type 2 diabetes is a growing global health concern and is caused by insulin resistance and beta-cell dysfunction [7]. Recent evidence suggests that both insulin secretion and resistance are heritable traits [8] and studies have also demonstrated a genetic contribution to defective insulin secretion and resistance in individuals with type 2 diabetes [9, 10].

Genome-wide association studies (GWAS) have revealed many genetic susceptibility loci for type 2 diabetes and related traits, such as insulin resistance and beta-cell dysfunction [10–15]. In addition, we have recently shown an association of the rs11708067 polymorphism in the *ADCY5* (adenylate cyclase 5) gene with increased 2-h glucose levels and decreased homeostasis model assessment of beta-cell function (HOMA2-B) in Swedish women with GDM [16].

Only a few studies have examined the genetic susceptibility to postpartum diabetes in women with previous GDM [4, 17]. In addition, the genetic architecture of postpartum diabetes and its related traits could differ between ethnicities. Thus, we sought to validate the association of single nucleotide polymorphisms (SNPs) from previous GWAS [10–15, 18] with indices of beta-cell function, insulin resistance, and eventual progression to diabetes in a cohort of women from Southern Sweden with a history of GDM. The association of genetic variation with a phenotype can be mediated by gene expression in the target tissues. Such loci, which can explain the variation in the mRNA levels, are referred to as expression quantitative trait loci (eQTLs) [19]. Therefore, we aimed to examine whether the eventual associated SNPs were also eQTLs in the RNA-Seq data from 191 human pancreatic donor islets [20] as well as insulin target tissues from the Genotype-Tissue Expression project (GTEx) [21].

## Methods

### Participants

Women delivering between 2003 and 2005 were invited to participate in the study in the County of Skane in Southern Sweden, as described previously [22]. GDM was diagnosed by a 75-g oral glucose tolerance test (OGTT) at the 28th and/or the 12th week of gestation for those with a first-degree relative with diabetes or previous GDM. Study participants were followed-up for development of diabetes using OGTT up to 5 years postpartum or until a diagnosis of diabetes; data were accessible through the primary care journals. GDM and diabetes were diagnosed according to the diagnostic criteria recommended by the WHO in 1999 [23]. Based on these criteria and the availability of stored DNA, a cohort of 374 women with previous GDM (57 of whom developed diabetes) were included in the present study.

### Glycaemic trait measurements

Women underwent OGTT with measurements of both glucose and insulin concentrations at 0, 30, and 120 min to calculate indices of beta-cell function and insulin resistance, at 1‒2 years postpartum, as reported previously [24]. Homeostasis model assessment of insulin resistance (HOMA-IR) was used to estimate insulin resistance (fasting serum insulin × fasting plasma glucose)/22.5 [25]. Insulin secretion capacity was estimated using the disposition index ([insulinogenic index (insulin _30 min_ – insulin _0 min_)/(glucose _30 min_ − glucose _0 min_)]/HOMA-IR) [26].

### Genotyping

DNA was extracted from whole blood using the MaxiPrep Kit (QIAGEN, Sollentuna, Sweden). SNPs were genotyped using a Sequenom massARRAY platform or TaqMan allelic discrimination assay with an ABI Prism 7900 sequence detection system (Applied Biosystems, Foster City, CA, USA). The success rate of genotyping was > 90%. Replication genotyping of 6% of the samples showed > 98% concordance. All SNPs were in Hardy–Weinberg equilibrium (HWE), except for rs11920090 and rs6467136, which significantly deviated in women who did not develop diabetes postpartum (*p* < 0.01), and were eventually excluded from the analysis. We analysed 12 SNPs previously shown to be associated with measures of insulin secretion [11, 12, 14], and 4 SNPs previously shown to be associated with measures of insulin resistance in GWAS [10, 11, 13], for association with disposition index and HOMA-IR, respectively, in women with previous GDM. We also analysed 70 (2 out of 72 were excluded for not being in HWE) SNPs, previously associated with diabetes in GWAS [11, 15, 18], for association with diabetes postpartum.

### Exploration of expression quantitative trait loci (eQTLs)

Polymorphisms associated with disposition index, HOMA-IR, or diabetes were assessed for association with gene expression in human pancreatic islets in RNA-Seq data of 191 donors [19] or insulin target tissues from GTEx [20]. The data are uploaded to EGA (https://ega-archive.org/) with the following accession numbers: RNAseq: EGAS00001004042, GWAS: EGAS00001004044, and Phenotype: EGAS00001004056.

### Statistical analyses

All statistical analyses were conducted using IBM SPSS Statistics for Windows, version 22.0 (Armonk, NY) and PLINK (version 1.09, http://pngu.mgh.harvard.edu/~purcell/plink/index.shtml). Categorical variables are shown as N. They are presented as mean ± standard deviation (SD). Student's *t*-test was used to test for differences between group means. Generalised linear models with maximum likelihood estimates were used for estimating SNP associations with disposition index and HOMA-IR. Logistic regression models were used for SNP associations with diabetes. Age, ethnicity, and body mass index (BMI) were used as covariates/confounders, and data are presented as beta estimates and standard errors (SE). Correction for multiple testing was performed using permutations. We applied a false discovery rate (FDR) for the association analysis of postpartum diabetes. Since it was a validation of previous associations, we considered *p* ≤ 0.05 as significant. The top 4 SNPs associated with the disposition index and the 4 SNPs tested for association with HOMA-IR were used to construct Genetic Risk Scores (GRS) [27] for their respective traits as well as for diabetes using equal weights.

## Results

### Clinical characteristics

Clinical characteristics of the study subjects were detailed previously [28]. Table 1 presents some of the relevant clinical characteristics of the studied women. The women who developed diabetes postpartum were older (Mean = 34.61, SD 4.76 vs. 32.56, SD 4.77, *p* = 0.003), had a higher BMI (Mean = 30.48, SD 6.15 vs. 24.29, SD 4.43, *p* < 0.0001), lower disposition index (Mean = 3.00, SD 6.62 vs. 9.00, SD 15.27, *p* = 0.0038), and higher HOMA-IR (Mean = 2.56, SD 3.32 vs. 0.34, SD 3.70, *p* < 0.0001) compared to those without postpartum diabetes.

**Table 1:** Clinical characteristics of the studied women

Variable	All women	Women with postpartum diabetes	Women without postpartum diabetes	*p* value#
Number of women	374	57	317	
Ethnicity [European/non-European/unknown] (N)	286/77/11	29/26/2	257/51/9	
Maternal age (years)	32.87 (4.82)	34.61 (4.76)	32.56 (4.77)	0.003
BMI (kg/m2)	25.24 (5.22)	30.48 (6.15)	24.29 (4.43)	< 0.0001
HOMA-IR*	0.68 (3.72)	2.56 (3.32)	0.34 (3.70)	< 0.0001
Disposition index*	8.09 (14.45)	3.00 (6.62)	9.00 (15.27)	0.0038
Fasting plasma glucose (mmol/L)*	5.23 (1.97)	6.32 (1.29)	5.03 (2.02)	< 0.0001
30 min plasma glucose (mmol/L)*	7.98 (3.11)	9.19 (3.87)	7.76 (2.91)	0.0013
120 min plasma glucose (mmol/L)*	6.56 (2.87)	9.17 (2.86)	6.09 (2.62)	< 0.0001

N = number. * Data from OGTT performed at 1–2 years postpartum. Data are presented as mean ± SD. Differences in means were tested using Student's *t*-test. # *p* value for differences between women with postpartum diabetes and women without postpartum diabetes

### Association of SNPs with disposition index

The risk A-allele of SNP rs11708067 in the *ADCY5* locus was associated with decreased disposition index (beta = − 0.90, SE 0.38, *p* = 0.019) after adjustment for age, BMI, and ethnicity (Table 2). Interestingly, the rs11708067 polymorphism was an eQTL for both *ADCY5* and the antisense transcript for *ADCY5* (Table 3). The *ADCY5* rs11708067 genotype was also associated with 2-h glucose level, though only in the additive model (AG vs. GG: beta = 0.62, SE 0.33, *p* = 0.06; AA vs. GG: beta = 0.71, SE 0.32, *p* = 0.027).

**Table 2:** Association of SNPs with disposition index at 1–2 years postpartum in women previous GDM

SNP	Locus	Location	CHR	N	EA	B (SE)	*p* value
rs11708067	*ADCY5*	Intron	3	358	A	− 0.90 (0.38)	0.019
rs340874	*PROX1*	Intergenic	1	305	C	0.09 (0.15)	0.56
rs560887	*G6PC2/ABCB11*	Intron	2	303	C	0.027 (0.13)	0.83
rs10885122	*ADRA2A*	Intergenic	10	313	G	− 0.075 (0.37)	0.84
rs4607517	*GCK*	Intergenic	7	340	A	− 0.005 (0.17)	0.98
rs2191349	*DGKB/TMEM195*	Intergenic	7	368	T	− 0.07 (0.20)	0.72
rs7034200	*GLIS3*	Intron	9	342	A	− 0.04 (0.19)	0.82
rs7944584	*MADD*	Intron	11	303	A	− 0.24 (0.28)	0.40
rs174550	*FADS1*	Intron	11	304	T	− 0.001 (0.13)	0.99
rs10830963	*MTNR1B*	Intron	11	314	G	0.12 (0.18)	0.49
rs11605924	*CRY2*	Intron	11	365	A	− 0.60 (0.32)	0.064
rs7756992	*CDKAL1*	Intron	6	313	G	− 0.25 (0.20)	0.21

SNP = single nucleotide polymorphism, CHR = chromosome, EA = effect allele, B = beta/effect size, SE = standard error; N = number of women with successful genotyping

**Table 3:** rs11708067 is an eQTL for *ADCY5* and the antisense transcript of *ADCY5* in islets

SNP	Gene	Gencode ID	Beta	t_STAT	*p* value
rs11708067	ADCY5_ANTISENSE TRANSCRIPT	ENSG00000272678	0.70	5.91	1.56e−08
rs11708067	ADCY5	ENSG00000173175	0.45	3.63	0.00037

SNP = single nucleotide polymorphism; Number of islets = 191

### Association of SNPs with HOMA-IR

The risk C-allele of the insulin receptor substrate-1 (*IRS1*) rs2943641 polymorphism showed a trend towards association with increased HOMA-IR (beta = 0.36, SE 0.18, *p* = 0.050) after adjustment for age, BMI, and ethnicity (Table 4). A search of this SNP in the GTEx database (from public data [https://gtexportal.org/]) [20] showed that this SNP is an eQTL for *IRS1* in the adipose tissue (normalised effect size in sub-cutaneous adipose tissue for the C allele = − 0.3, *p* = 1–4^e−16^ and normalised effect size in visceral adipose tissue for the C allele = − 0.23, *p* = 6.1^e−12^).

**Table 4:** Association of SNPs with HOMA-IR at 1–2 years postpartum in women with previous GDM

SNP	Locus	Location	CHR	N	EA	B (SE)	*p* value
rs2943641	IRS1	Intergenic	2	313	C	0.36 (0.18)	0.050
rs4675095	IRS1	Intron	2	355	A	− 0.11 (0.55)	0.83
rs1801282	PPARG	Coding – missense	3	307	C	0.29 (0.41)	0.48
rs35767	IGF1	Near Gene-5	12	305	G	0.02 (0.31)	0.94

SNP = single nucleotide polymorphism, CHR = chromosome, N = number of women with successful genotyping, EA = effect allele, B = beta/effect size, SE = standard error; HOMA-IR was normalised using rank normal transformation

### Association studies of diabetes postpartum

The T-allele of rs4607103, near the ADAM metallopeptidase with thrombospondin type 1 motif 9 (*ADAMTS9*) gene, was associated with an increased risk of diabetes postpartum (OR for the C-allele 0.47 (CI: 0.30–0.73), *p* = 0.00055; pFDR = 0.039) (Table 5). The CC carriers have more insulin resistance than TT carriers (beta = − 0.11, SE = 0.05, *p* = 0.036).

**Table 5:** SNPs with nominal p-values for association with diabetes up to 5 years after a pregnancy complicated by GDM

SNP	Gene/nearest gene	Location	CHR	RA/OA	OR (CI)	*p* value	*p* value (FDR_BH)
rs4607103	ADAMTS9-AS2	Intron	3	C/T	0.47 (0.30–0.73)	0.00055	0.039
rs4607517	GCK	Intergenic	7	A/G	1.765 (1.09–2.85)	0.019	0.4902
rs1552224	CENTD2	Intergenic	11	A/C	2.22 (1.09–4.61)	0.024	0.4902
rs11634397	ZFAND6	Intergenic	15	G/A	1.58 (1.03–2.42)	0.037	0.4902
rs7578597	THADA	Coding—missense	2	T/C	3.29 (1.004–10.75)	0.037	0.4902
rs4457053	ZBED3	Intron of ZBED3-AS1	5	G/A	1.56 (1.02–2.38)	0.041	0.4902

SNP = single nucleotide polymorphism, CHR = chromosome, SNP = single nucleotide polymorphisms, RA/OA = risk allele/other allele, OR = estimated odds ratio (for risk), *P* value = nominal p-value for this test, FDR_BH = Benjamini & Hochberg (1995) step-up FDR control

### Association studies of generic risk scores (GRSs)

The GRS of the top four SNPs (*ADCY5* rs11708067, *MADD* rs7944584, *CRY2* rs11605924, and *CDKAL1* rs7756992) among 12 SNPs tested for association with the disposition index (Table 2) [29–32] using equal weights was associated with the disposition index (beta = − 0.31, SE = 0.29, p [adjusted for age, BMI, and ethnicity] = 0.00096; p for diabetes [no covariates] = 0.81).

The GRS of the four SNPs studied for association with HOMA-IR (*IRS1* rs2943641, *IRS1* rs4675095, *PPARG* rs1801282, and *IGF1* rs35767) (Table 4) [10, 11, 33, 34] using equal weights showed an association with HOMA-IR (beta = 1.13, SE = 0.48, p [adjusted for age, BMI, and ethnicity] = 9.72874e−11; p for diabetes [no covariates] = 0.63).

## Discussion

In this study, we examined the genetic susceptibility of the genetic risk loci from previous GWAS with disposition index, HOMA-IR, and the development of postpartum diabetes in women with previous GDM from Southern Sweden. Women with previous GDM who developed postpartum diabetes had a higher HOMA-IR and a lower disposition index compared to those without postpartum diabetes. We found an association between the risk A-allele of *ADCY5* rs11708067 and decreased disposition index derived from OGTT at 1–2 years postpartum in women with previous GDM. This was concordant with previous findings of an association of rs11708067 with HOMA-B, a measure of insulin secretion [11]. *ADCY5* encodes adenylate cyclase 5, which catalyses the generation of cAMP. When glucagon-like peptide 1 (GLP-1) binds to its receptor in the pancreatic beta cells, it induces cAMP-mediated activation of protein kinase A, transcription of the proinsulin gene, and secretion of insulin [35]. Previous studies reported reduced *ADCY5* mRNA expression in islets due to risk alleles at rs11708067 [29] and suggested that *ADCY5* rs11708067 is essential for coupling glucose to insulin secretion in human islets [29]. Wagner et al. implicated the rs11708067 polymorphism in defective proinsulin-to-insulin conversion [36]. Furthermore, rs11708067 is an eQTL in islets for both *ADCY5* and the antisense transcript for *ADCY5* supports the role of this SNP in impairment of insulin secretion in women with previous GDM, thus increasing the risk of diabetes postpartum. We also found that the *ADCY5* rs11708067 genotype was associated with 2-h glucose level, consistent with the previous results from our group [16] as well as others [11, 36].

Our analysis also identified an association of the risk C-allele of rs2943641 near the *IRS1* gene with HOMA-IR in our cohort. *IRS1* encodes a member of the IRS protein substrate family. IRS1 is a substrate of the insulin receptor tyrosine kinase, which plays a crucial role in the insulin signalling pathway and is expressed in insulin-sensitive tissues [37]. This finding agrees with an earlier study by Rung et al., who reported an association of the C-allele of rs2943641 with measures of insulin resistance (HOMA-IR and Insulin sensitivity index [ISI]) and hyperinsulinemia in French, Danish, and Finnish participants from population-based cohorts [10]. Moreover, this SNP has been associated with type 2 diabetes in meta-analyses involving European [10] and Japanese participants [38]. Notably, rs2943641 is an eQTL for the *IRS1* gene in adipose tissue. Thus, this genetic variant near *IRS1* may increase the risk of postpartum diabetes in women with previous GDM through increased insulin resistance.

We have shown that the T-allele of rs4607103, near the *ADAMTS9* gene, could predict development of diabetes in our cohort. However, the risk allele in this study was opposite to that reported for diabetes [15]. ADAMTS9 is a member of the ADAMTS (a disintegrin and metalloproteinase with thrombospondin motifs) protein family. It is highly expressed in various tissues and is abundantly expressed in the heart and skeletal muscle [39]. Graae et al. showed that the C-allele was associated with increased expression of secreted ADAMTS9 and decreased insulin sensitivity and signalling in human skeletal muscle [40]. In contrast, in our data CC carriers seemed to have reduced insulin resistance compared to TT carriers, although there was no significant association. This phenomenon is not uncommon in genetics [41]; the actual functional variant in the *ADAMTS9* region is not yet known, and the rs4607103 polymorphism may be in a linkage disequilibrium with the functional variant in this region. Interestingly, the C-allele was associated with protection from type 2 diabetes in African Americans [42], but our study population was primarily European. Moreover, a potential gender effect could not be excluded [43]. We do not have any data on the presence of antibodies in women who developed diabetes postpartum. Since approximately 1% of women with diabetes postpartum are diagnosed with type 1 diabetes in Sweden [44], the vast majority of women included in this study were expected to have been diagnosed with type 2 diabetes.

Finally, we also tested the association of GRS with insulin secretion and resistance as well as postpartum diabetes. A GRS is an estimate of the cumulative contribution of genetic factors to a specific outcome of interest in an individual [27]. In our study, the GRS for disposition index and HOMA-IR was constructed from 4 SNPs associated with their respective traits. The GRS predicted variations in both traits. However, these GRS were not independently associated with diabetes, which could potentially be attributed to low statistical power. Recent studies showed heterogeneity of type 2 diabetes and GDM with insulin secretion driven subtypes being different compared to insulin resistance driven subtypes [45–47]. The lack of association could also be attributed to this potential heterogeneity which would require further research. Overall, the results of this study illustrate the crucial role of defective insulin secretion and impaired insulin sensitivity in women with previous GDM who develop postpartum diabetes, and how genetic risk factors could be used to identify these women. Our results and potential future studies in other populations might help to identify clinical and genetic risk profile(s) that could provide an early and appropriate preventive strategy for this high-risk group.

A strength of this study was the use of the disposition index, derived from OGTT, as a measure of beta-cell function adjusted for insulin resistance. The women in the study were followed up for development of diabetes up to 5 years postpartum. Our study provides novel insights into the genetic variants associated with postpartum diabetes and its related traits. A major weakness of the study was the failure to correct for multiple comparisons in the analyses, except for the analysis of development of diabetes after pregnancy. However, the analyses were adjusted for age, BMI, and ethnicity; permutations were performed to address this issue to a certain extent. The studied SNPs were previously shown to be associated with their respective traits, suggesting that this study could be considered a replication study. The study did not aim to detect small potential effects of the studied SNPs on insulin secretion and sensitivity.

## Conclusions

The current study demonstrates the genetic susceptibility for impaired insulin secretion and sensitivity, as well as for the development of diabetes in women with a history of GDM. This finding could aid in the early identification of women at higher risk of developing diabetes postpartum.

## Data Availability

The datasets used and/or analysed during the current study are available from the corresponding author on reasonable request.

## Abbreviations

*ADAMTS9*: ADAM metallopeptidase with thrombospondin type 1 motif 9 gene
*ADCY5*: Adenylate cyclase 5 gene
BMI: Body mass index
eQTL: Expression quantitative trait loci
FDR: False discovery rate
GDM: Gestational diabetes mellitus
GLP-1: Glucagon-like peptide 1
GRS: Genetic risk score
GTEx: Genotype-Tissue Expression project
GWAS: Genome-wide association studies
HOMA2-B: Homeostasis model assessment of beta-cell function
HOMA-IR: Homeostasis model assessment of insulin resistance
*IRS1*: Insulin receptor substrate 1
ISI: Insulin sensitivity index
OGTT: Oral glucose tolerance test
SNP: Single nucleotide polymorphisms
